# Suplementação de L-Carnitina no Coração Diabético

**DOI:** 10.36660/abc.20210717

**Published:** 2021-10-06

**Authors:** Filipe Welson Leal Pereira, Sergio Alberto Rupp de Paiva

**Affiliations:** 1 Faculdade de Medicina de Botucatu Universidade Estadual Paulista BotucatuSP Brasil Faculdade de Medicina de Botucatu, Universidade Estadual Paulista (Unesp), Botucatu, SP – Brasil

**Keywords:** Carnitina, Metabolismo Energético, Reação em Cadeia da Polimerase

A carnitina é um nutriente não essencial, derivado de aminoácidos, e sua principal fonte alimentar são a carne vermelha, a carne de aves e os laticínios. Parte da carnitina pode ser produzida endogenamente a partir da lisina e da metionina, principalmente pelo fígado e rins.^1^ Age sobretudo como cofator enzimático para o transporte de ácidos graxos de cadeia longa do citoplasma para o interior da mitocôndria e subsequente degradação para betaoxidação, e trata-se de uma via muito importante para metabolismo energético. Dessa forma, a carnitina é essencial como combustível para o músculo, e mais de 95% da substância é encontrada no músculo esquelético.^1^ Fígado, coração, cérebro e rins detêm o restante das reservas de carnitina corporal ou possuem condições para síntese,^1^ e tal distribuição mostra a importância da carnitina nesses órgãos. Estudos sobre a suplementação de L-carnitina estão sendo desenvolvidos na sarcopenia,^2^ em doenças hepáticas,^3^ na insuficiência cardíaca^4^ e em doenças renais^5^ e neurológicas.^6 , 7^

A maioria dos estudos mostrou benefício da L-carnitina nos fatores de risco cardiovascular, e sua suplementação reduz hipertensão, hiperlipidemia, hiperglicemia, diabetes melito insulinodependente, resistência à insulina, obesidade, inflamação e estresse oxidativo.^4 , 8 - 12^

Além de atenuar os fatores de risco para a aterosclerose, a suplementação de L-carnitina pode melhorar o metabolismo energético do coração “diabético”, por meio da manipulação dos grupos acetila e acila pela mitocôndria. Desde que a L-carnitina é responsável por sua transferência através da membrana mitocondrial.^13^ Outro mecanismo proposto é o de que a suplementação de L-carnitina pode melhorar o microambiente inflamatório e oxidativo cardíaco causado pela hiperglicemia.^14^ Nesse sentido, um artigo de revisão sistemática e a metanálise de ensaios clínicos randomizados mostraram que a suplementação de L-carnitina foi associada a redução de PCR, (interleucina 6 (IL-6), fator de necrose tumoral α (TNF-α) e malondialdeído no plasma, e ao aumento das concentrações plasmáticas de superóxido dismutase.^9^

No que diz respeito à obesidade, observamos em revisão sistemática que a suplementação de L-carnitina reduziu peso corporal, índice de massa do corpo (IMC) e massa gorda, e a análise de subgrupo baseada na faixa de IMC basal dos participantes mostrou mais reduções em adultos com sobrepeso ou obesidade do que nos indivíduos com faixa de IMC normal.^12^

O artigo publicado na edição dos Arquivos Brasileiros de Cardiologia tem como base obesidade, diabetes e inflamação.^15^

Os autores apresentaram resultados de estudo experimental, em que camundongos obesos com diabetes induzido receberam L-carnitina. Foram avaliadas a proteína da quemerina e o receptor CMKLRI (do inglês *chemokine like receptor 1* ) no soro e nos tecidos cardíaco e adiposo, outros marcadores inflamatórios além da resistência insulínica.^15^

A quemerina é uma nova adipocina que participa dos estágios iniciais da inflamação aguda e participa no desenvolvimento de hipertensão, progressão de lesões ateroscleróticas que possivelmente age por meio de seu receptor CMKLR1.^16^ Os autores conseguiram mostrar associação entre o consumo de L-carnitina e a redução dos valores de quemerina sérica nos tecidos cardíaco e adiposo em camundongos diabéticos tratados, além de reduzir valores de outros marcadores inflamatórios (IL 1β e TNF- α) após 4 semanas do tratamento. Observou-se, também, que o grupo que sofreu a intervenção apresentou melhor perfil de resistência insulínica.^15^

A redução da quemerina e de outros marcadores inflamatórios podem estar mostrando a importância do processo inflamatório no coração diabético; entretanto, ainda é cedo para recomendar a suplementação de L-carnitina. Existem estudos que mostram que produtos de degradação da carnitina pela microbiota intestinal podem gerar o N-óxido-trimetilamina (TMAO).^17^ Observou-se em revisão sistemática que o TMAO esteve associado, em modo “dose-dependente”, ao aumento de eventos cardiovasculares e mortalidade.^17^ Dessa maneira, faltam estudos que levem a entendimento do papel da suplementação da L-carnitina no tratamento adjuvante para a situação do coração diabético.
