# Supplementary material for: Suplementação de L-Carnitina no Coração Diabético
Source: Arq Bras Cardiol. 2021 Oct 6;117(4):726–7. [Article in Portuguese] doi: 10.36660/abc.20210717 (PMC8528375; doi:10.36660/abc.20210717)
Supplement: Translation

# Translation: L-Carnitine Supplementation in the Diabetic Heart

**Authors:** Filipe Welson Leal Pereira, Sergio Alberto Rupp de Paiva

**Affiliations:** 1 Brazil Faculdade de Medicina de Botucatu, Universidade Estadual Paulista (Unesp), Botucatu, SP – Brazil

**Keywords:** Carnitine, Energy Metabolism, Polymerase Chain Reaction

Carnitine is a non-essential nutrient, which is derived from amino acids. The main food source of carnitine is red meat, poultry and dairy products. Part of the carnitine can be produced endogenously, from lysine and methionine mainly by the liver and kidneys.^1^ It acts primarily as an enzymatic cofactor for the transport of long-chain fatty acids from the cytoplasm into the mitochondria, and subsequent degradation to beta-oxidation, being a very important energy metabolism pathway. Therefore, carnitine is essential as fuel for muscles, and more than 95% of the total body carnitine is found in skeletal muscle.^1^ The liver, heart, brain and kidneys have the remainder of the body’s carnitine reserves or are able to synthesize it.^1^ This distribution shows the importance of carnitine in these organs. L-carnitine supplementation has been studied in sarcopenia,^2^ in liver diseases,^3^ in heart failure,^4^ in kidney diseases^5^ and in neurological diseases.^6 , 7^

Most studies have shown the benefit of L-carnitine on cardiovascular risk factors. L-carnitine supplementation reduces hypertension, hyperlipidemia, hyperglycemia, insulin-dependent diabetes mellitus, insulin resistance, obesity, inflammation and oxidative stress.^4 , 8 - 12^

In addition to attenuating risk factors for atherosclerosis, L-carnitine supplementation can improve the energy metabolism of the “diabetic” heart. The improvement can occur by normalizing the manipulation of the acetyl and acyl groups by the mitochondria, as L-carnitine is responsible for their transfer through the mitochondrial membrane.^13^ Another proposed mechanism is that L-carnitine supplementation can improve the inflammatory and oxidative cardiac microenvironment caused by hyperglycemia.^14^ Thus, a systematic review article and meta-analysis of randomized clinical trials showed that L-carnitine supplementation was associated with a reduction in the levels of CRP, Interleukin-6 (IL-6), tumor necrosis factor-α (TNF-α) and plasma malondialdehyde, and increased plasma levels of superoxide dismutase.^9^

Regarding obesity, a systematic review showed that L-carnitine supplementation reduced body weight, body mass index (BMI) and fat mass, and the subgroup analysis based on the participants’ basal BMI range showed more reductions in overweight or obese adults than in those within the normal BMI range.^12^

The article published in this issue of the *Arquivos Brasileiros de Cardiologia* is based on obesity, diabetes and inflammation.^15^

The authors presented results of an experimental study, in which obese mice with induced diabetes received L-carnitine. Chemerin protein and Chemokine-like Receptor 1 (CMKLR1) levels in serum, cardiac and adipose tissue, as well as other inflammatory markers were evaluated, in addition to insulin resistance.^15^

Chemerin is a new adipokine that participates in the early stages of acute inflammation and participates in the development of hypertension, progression of atherosclerotic lesions, possibly acting through its CMKLR1 receptor.^16^ The authors were able to demonstrate an association between L-carnitine consumption and a reduction in serum chemerin values and in cardiac and adipose tissue in treated diabetic mice, in addition to a reduction in the levels of other inflammatory markers (IL-1β and TNF-α) after 4 weeks of treatment. They also observed that the group submitted to the intervention had a better insulin resistance profile.^15^

The decrease in the levels of chemerin and other inflammatory markers may be showing the importance of the inflammatory process in the diabetic heart. However, it is still too early to recommend L-carnitine supplementation. Some studies have shown that carnitine degradation products by the intestinal microbiota can generate Trimethylamine N-oxide (TMAO).^17^ A systematic review showed that TMAO was associated with an increase in cardiovascular events and mortality in a dose-dependent manner.^17^ Thus, more studies are necessary to understand the role of L-carnitine supplementation in the adjuvant treatment of the diabetic heart.
